# The Examination of the Role of Rice Lysophosphatidic Acid Acyltransferase 2 in Response to Salt and Drought Stresses

**DOI:** 10.3390/ijms23179796

**Published:** 2022-08-29

**Authors:** Aamir Ali Shaikh, Alfatih Alamin, Chenxi Jia, Wei Gong, Xianjun Deng, Qingwen Shen, Yueyun Hong

**Affiliations:** National Key Laboratory of Crop Genetic Improvement, Huazhong Agricultural University, Wuhan 430070, China

**Keywords:** abscisic acid response, lysophosphatidic acid acyltransferase, osmotic stress, phosphatidic acid, rice (*Oryza sativa*)

## Abstract

Phosphatidic acid (PA) is an important signal molecule in various biological processes including osmotic stress. Lysophosphatidic acid acyltransferase (LPAT) acylates the sn-2 position of the glycerol backbone of lysophosphatidic acid (LPA) to produce PA. The role of LPAT2 and its PA in osmotic stress response remains elusive in plants. Here we showed that LPAT2-derived PA is important for salt and drought stress tolerance in rice. Rice LPAT2 was localized to the endoplasmic reticulum (ER) to catalyze the PA synthesis. The *LPAT2* transcript was induced by osmotic stress such as high salinity and water deficit. To reveal its role in osmotic stress response, an *LPAT2* knockdown mutant, designated *lpat2*, was isolated from rice, which contained a reduced PA level relative to wild type (WT) plants under salt stress and water deficit. The *lpat2* mutant was more susceptible to osmotic stress and less sensitive to abscisic acid (ABA) than that of WT, which was recovered by either PA supplementation or genetic *LPAT2* complementation. Moreover, suppressed LPAT2 also led to a large number of differentially expressed genes (DEGs) involved in diverse processes, particularly, in ABA response, kinase signaling, and ion homeostasis in response to salt stress. Together, LPAT2-produced PA plays a positive role in osmotic tolerance through mediating ABA response, which leads to transcriptional alteration of genes related to ABA response, protein kinase signaling, and ion homeostasis.

## 1. Introduction

Osmotic stress, mostly imposed by drought and high salinity, is the most adverse factor that inhibits plant growth, development, and crop yield, and hence reduces agricultural productivity globally [1,2,3,4]. Plants have developed different mechanisms to adapt to environmental stresses [4,5,6,7,8]. To protect cells from damage by osmotic stress, plants must sense environmental stimuli and trigger the response to proceed the signal transduction, resulting in the changes of protein activity, subcellular localization, gene expression, which thus causes physiological, metabolic, and morphological alterations to deal with adverse environment [4,5,6,7,8,9,10]. Of these mechanisms, sensing environmental stimuli is an early and important event. However, our knowledge about molecular mechanisms in the early event of signaling involved in osmotic stress is still limited.

Abscisic acid (ABA) is a key phytohormone involved in stomatal closure to reduce water loss in response to osmotic stress including salt and drought stresses [2,10]. It also plays important roles in seed maturation, seed germination, and flowering [11]. A higher physiological level of ABA under osmotic stress has been shown to regulate signal cascades in which protein kinases are involved [12]. For example, high saline conditions guide the cells to produce more endogenous ABA, leading to stomatal closure to reduce water loss [2]. The ABA receptors PYR (pyrabactin resistance) and PYL (pyrabactin resistance-like) have been identified in Arabidopsis [13,14]. Two components, sucrose nonfermenting (SNF1)-related protein kinase 2 (SnRK2) and protein phosphatase (PP2C, also named as ABI1), are the hallmarks exhibiting opposite effects in ABA signaling under osmotic stress [12,15]. In the presence of ABA, SnRK2 is activated by autophosphorylation to phosphorylate the downstream substrates, such as ABA responsive element-binding transcription factor (AREB/ABF) to regulate gene expression [15], and an anion channel (SLOW ANION CHANNEL-ASSOCIATED1; SLAC1) involved in stomatal closure [16], thus enhances osmotic stress tolerance. By contrast, PP2C (ABI1) is a negative regulator through the dephosphorylation of SnRK2 to block its kinase activity in ABA signaling [17]. 

Phosphatidic acid is a critical phospholipid molecule with anionic and small headgroup [18], functioning as not only an intermediate for glyceroliplid biosynthesis but also an important signal molecule in various biological processes, particularly osmotic stress in plants [19,20]. A small change in PA level may result in a substantial effect due to its function in the upstream of signaling cascades [20]. Phosphatidic acid with various species of acyl group in specific membrane region has been shown to bind to various effectors involved in diverse cellular processes in plants [19,20,21]. In plants, PA can be generated through three major routes [22]. First, PA is assembled de novo via two steps of acylation at the sn-1 and sn-2 hydroxyl groups of glycerol-3-phosphate, which is catalyzed sequentially by glycerol-3-phosphate acyltransferase (GPAT) and lysophosphatidic acid acyltransferase (LPAT) by using acyl-CoA in the endoplasmic reticulum (ER), or acyl–acyl carrier protein (acyl-ACP) in plastids in plants [23]. Second, PA can be directly derived from phospholipids such as phosphatidylcholine (PC) hydrolysed by phospholipase D (PLD) [20]. Third, PA can also be generated through the combined actions of phospholipase C (PLC) and diacylglycerol kinase (DGK) by using phospholipids as substrates [19,24]. 

LPAT catalyzes the acylation at the sn-2 hydroxyl group of glycerol backbone of lysophosphatidic acid (LPA) to produce PA, which is responsible for PA de novo synthesis. Arabidopsis contains five LPATs, LPAT1-5 [25,26,27]. LPAT1 is a chloroplast-localized enzyme involved in the prokaryotic pathway [25], whereas LPAT2-5 are the ER-localized enzymes and may be involved in the eukaryotic pathway of glycerolipid synthesis [26,27]. The activities of LPAT1 and LPAT2 were higher, whereas the activities of LPAT3, 4, and 5 were relatively lower under the conditions tested [25,26]. The knockout of *LPAT1* leads to embryo lethality at the heart or torpedo development stages [25,28], whereas loss of LPAT2 results in female gametophytic lethality in Arabidopsis [26]. Overexpression of *LPAT2* promotes root growth in response to phosphate starvation, as compared to WT in Arabidopsis [29]. LPAT4 and LPAT5 are functionally redundant in nitrogen starvation response [27]. Phosphatidic acid is predominantly synthesized de novo in the eukaryotic pathway occurring in the ER. The lethality of the *LPAT2* knockout mutant suggests that LPAT2 is a primary and essential LPAT isoform for PA de novo synthesis.

Among PA sources, PLD-derived PA was extensively studied and found to be important in salt and drought stresses [30,31,32,33,34,35,36]. However, the role of the de novo synthesized PA in osmotic stress remained unknown due to the lethality of the primary isoforms of LPAT family in plants. Moreover, rice is a staple crop for almost half of the global population and also an important model monocotyledon plant species for genomic studies [37]. To date, none of the rice LPATs has been functionally characterized. We have recently isolated an *LPAT2* knockdown mutant from rice, which can be used to study for its role in the post-embryo stages in response to osmotic stress. In this study, PA produced by LPAT2 was found to play a positive role in osmotic stress tolerance in rice. 

## 2. Results

### 2.1. Identification and Expression Pattern of Rice LPAT2 and Isolation of LPAT2 Knockdown Mutant

Based on the conserved domain structure and the sequence similarity to yeast, algae, and Arabidopsis LPATs, rice genome contains five putative LPATs, including OsLPAT1, 2, 3, 4, and 5 with conserved I [NH(X_4_)D], II, III, and IV motifs related to acyltransferase in various species (Supplementary Figure S1). The phylogenetic analysis showed that rice OsLPATs are classified into three clades. Rice OsLPAT1 (Os10g0497100), OsLPAT3 (Os04g0625200), and Arabidopsis AtLPAT1 (At4g30580) are classified into the same clade with yeast LPAT (ScLPAT0231) (Supplementary Figure S2), suggesting that rice OsLPAT1 and OsLPAT3 are more closely related to Arabidopsis LPAT1. Rice OsLPAT4 (Os05g0502200), OsLPAT5 (Os01g0782500), Arabidopsis AtLPAT4 (At1g75020), and AtLPAT5 (At4g18850) are classified into the same group containing algal LPAT (NoLPATU6U0). Rice OsLPAT2 (Os11g0637800) is grouped into the same clade with Arabidopsis AtLPAT2 (At3g57650) and AtLPAT3 (At1g51260) with more distantly related to other LPATs within the clade (Supplementary Figure S2).

**Figure 1:**
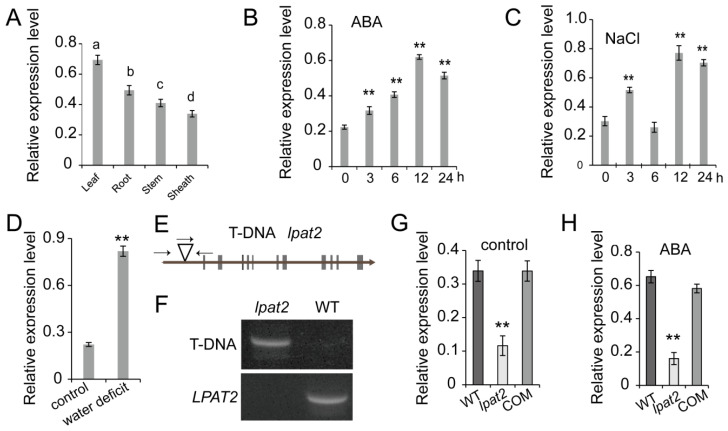
Rice *LPAT2* expression pattern, the *lpat2* mutant isolation, and genetic complementation. (**A**) The *LPAT2* expression level in various tissues. Values are means ± SD (*n* = 3). (**B**–**D**). The *LPAT2* transcript level in leaves in response to ABA. The different letters indicate the significant difference at *p* < 0.05 based on the Duncan’s multiple ranges. (**B**), salt stress (**C**), and water deficit (**D**) treatments. Total RNA was extracted from leaves of two-week-old plants at different time points (0, 3, 6, 12, and 24 h) for 100 mM NaCl and 10-μM ABA treatments. For water deficit, the leaves were sampled from plants grown under well-watered conditions (control) or under water deficit (25 to 30% of soil water capacity) when watering were withheld for five days. Values are means ± SD (*n* = 3). (**E**) The T-DNA insertion site in rice *LPAT2*. The arrows indicate the locations of primers for T-DNA insertion by PCR. (**F**) The *lpat2* mutant isolation by PCR. (**G**,**H**) The *LPAT2* expression level in the *lpat2* mutant, wild type (WT) and *LPAT2* complementation (COM) plants under the control conditions (**G**) and ABA treatment (**H**). Data are means ± SD (*n* = 3). Two-week-old plants were treated with 10 μM ABA for 12 h. The *LPAT2* transcript level was determined by RT-qPCR by using *β-Actin* as an internal standard. ** indicates significant difference at *p* < 0.01 compared to WT plants based on Student’s *t* test.

Spatial and temporal regulation is important to all signaling events, which is particularly critical to lipid messengers because of their limited mobility. To investigate the tissue distributions of *LPAT2* transcript, the total RNA was extracted from various tissues for the expression analysis by RT-qPCR. The *LPAT2* mRNA was expressed in different tissues including leaf, root, stem, and sheath with the highest level in the leaf (Figure 1A). The *LPAT2* expression was dramatically induced by ABA treatment and salt stress, with the highest 12 h after treatment with ABA and NaCl, as compared to control plants without treatment (Figure 1B,C). The *LPAT2* transcription level was also highly induced by water deficit being three-fold higher than that of control conditions (Figure 1D). These results suggest that *LPAT2* is induced by osmotic stress and may be involved in osmotic stress response.

The role of LPAT2 and its PA in osmotic stress response remains elusive due to the *LPAT2* knockout mutant being lethal at the female gametophytic developmental stage [26]. To further characterize the role of LPAT2 in post-embryonic stages, a knockdown mutant, designated as *lpat2*, was isolated in rice in this study (Figure 1E–G). The *lpat2* mutant was caused by a T-DNA insertion in the *LPAT2* promoter region located 358 bp upstream of the start codon (Figure 1E,F). The *LPAT2* transcript level in the *lpat2* mutant was substantially lower than that of WT, with ~40% of WT (Figure 1G). The results suggest that *lpat2* is a knockdown mutant with a significantly lower level of *LPAT2* mRNA relative to WT. Moreover, the *LPAT2* mRNA level of the *lpat2* mutant was 25% of WT in response to ABA treatment due to less induction in the *lpat2* mutant relative to WT (Figure 1H).

### 2.2. LPAT2 Enhances Plant Tolerance to Osmotic Stress

**Figure 2:**
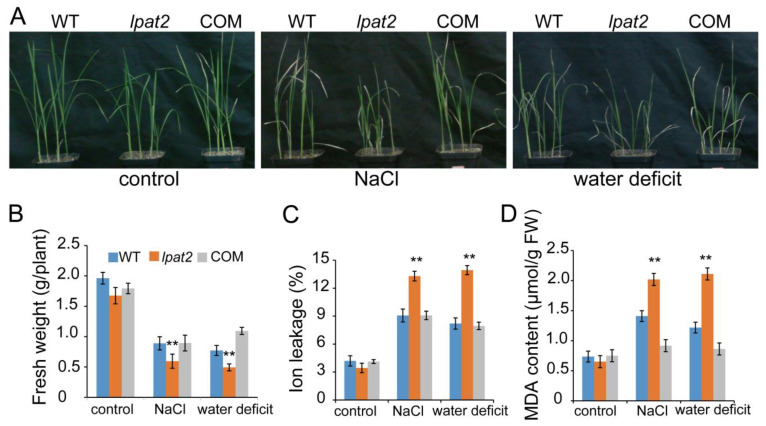
Suppressed LPAT2 led to reduced tolerance to salt and drought stresses. (**A**) Seedling growth phenotypes under the control, salt stress, and water deficit conditions. Three-week-old plants were treated with salt stress (150 mM NaCl) for 12 days, or water deficit (stopped watering for 10 days until leaves of wild type (WT) plants began wilting when the soil water content was approximately 11%). (**B**) The shoot fresh weight of plants shown in (**A**). Values are means ± SD (*n* = 10) from one of three independent experiments. (**C**) Ion leakage in leaves. Values are means ± SD (*n* = 3). (**D**) Malonaldehyde (MDA) content in leaves. Values are means ± SD (*n* = 3). Control, plants without stress treatment; COM, *LPAT2* genetic complementation. ** indicates significant difference at *p* < 0.01 compared to WT plants based on Student’s *t* test.

To determine whether LPAT2 is involved in osmotic stress, four-leaf-stage seedlings of the *lpat2* mutant and WT were transferred to soil and treated with NaCl stress and water deficit after one week of recovery. Under control conditions grown in soil with regular watering, the *lpat2* mutant exhibited a similar phenotype with WT (Figure 2A). However, when plants were treated with 150 mM NaCl, the shoot fresh weight of *lpat2* mutant plants was decreased by ~20%, as compared to WT (Figure 2B). The *lpat2* mutant plants were more damaged in response to salt stress, exhibiting higher malondialdehyde (MDA) content and increased ionic leakage relative to that of WT (Figure 2C,D). Likewise, the *lpat2* mutant was also more susceptible to water deficit with decreased shoot fresh weight and increased cellular damage (Figure 2). During the course of water deficit, the *lpat2* mutant seedlings wilted faster than WT, only 32% of the *lpat2* mutant seedlings were recovered after 7 days of water deficit treatment followed by re-watering, whereas 59% of WT seedlings were recovered.

To confirm the role of *LPAT2* in osmotic stress, the full length of the *LPAT2* codon sequence under the control of its native promoter was introduced into the *lpat2* mutant to generate genetic *LPAT2* complementation (COM) plants. The *LPAT2* transcript level in the COM plants was significantly higher than that of the *lpat2* mutant and comparable to that of WT plants as confirmed by RT-qPCR (Figure 1G). The sensitivity to salt and drought stresses in the *lpat2* mutant was rescued by genetic *LPAT2* complementation to the WT phenotype. The shoot fresh weight, ionic leakage, and MDA content in COM plants were almost similar to those in WT (Figure 2). These results suggest that LPAT2 plays a positive role in osmotic stress tolerance.

### 2.3. LPAT2 Enhances ABA Response 

**Figure 3:**
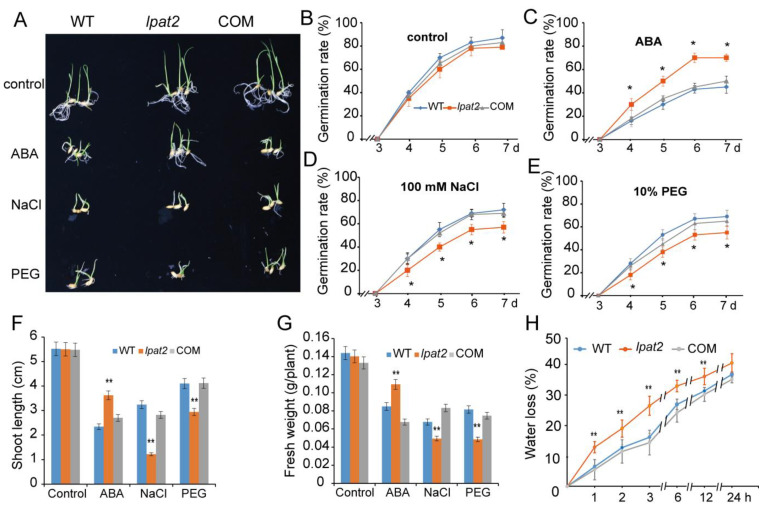
Suppressed LPAT2 led to less sensitivity to ABA and more sensitivity to osmotic stress in seed germination and seedling growth. (**A**–**E**) The seed germination rate in response to ABA and osmotic stress. The seeds were germinated in various conditions including the control, 10 μM ABA, 100 mM NaCl, and 10% polyethylene glycol (PEG) treatments. One hundred seeds per genotype were measured in each experiment. Values are means ± SD (*n* = 3). (**F**,**G**) Shoot length and shoot fresh weight of seedlings in response to ABA and osmotic stress. Four-day-old seedlings were transferred to small plastic cups without supplement (control), or with 10 μM ABA, 100 mM NaCl, and 10% PEG. Values are means ± SD (*n* = 15) from one representative of three independent experiments. (**H**) Water loss in leaves of rice plants. Three-week-old plants were removed from soils and left in ambient conditions. The water loss in leaves was expressed as the percentage of decrease in leaf fresh weight. Values are means ± SD (*n* = 3) from one of three independent experiments. * and ** indicate significant differences at *p* < 0.05, and *p* < 0.01, respectively, compared to the wild type (WT) based on Student’s *t* test.

Abscisic acid is an important phytohormone playing a positive role in osmotic stress tolerance via reduced water transpiration under osmotic stress [2,10]. To determine whether the involvement of LPAT2 in osmotic stress is mediated by ABA response, the seed germination was compared among WT, *lpat2*, and COM in response to ABA treatment. The germination rate of the *lpat2* mutant was not significantly different from that of WT and COM under control conditions without ABA treatment (Figure 3A,B). In the presence of 10 μM ABA, the seed germination rate in the *lpat2* mutant was significantly higher than that of WT. The germination rate of the *lpat2* mutant was ~70% on the sixth day of the ABA treatment, whereas that of WT seeds was ~37% only (Figure 3C). The results suggest that the *lpat2* mutant seeds were less sensitive to ABA compared to WT. Upon exposure to salt stress, an opposite phenotype of the *lpat2* mutant was found with a lower germination rate than that of WT (Figure 3D). A similar tendency was found when seeds were treated with 10% PEG (Figure 3E). Similarly, the *lpat2* mutant seedlings were less sensitive to ABA treatment than that of WT, exhibiting less inhibited growth in the *lpat2* mutant with longer shoots and greater shoot fresh weight than that of WT in the presence of ABA (Figure 3F,G). The less sensitivity to ABA in the *lpat2* mutant was rescued to WT phenotype by *LPAT2* complementation (Figure 3). By contrast, the *lpat2* mutant seedlings were more sensitive to osmotic stress, with substantial reductions of shoot length and fresh weight, as compared to WT and COM under salt stress and polyethylene glycol (PEG) treatments (Figure 3F,G). Moreover, the *lpat2* mutant leaves increased water loss compared to WT and COM under dehydration condition when plants were removed from soil in various time points (Figure 3H). The results suggest that LPAT2 enhances ABA sensitivity and plays a positive role in osmotic stress tolerance.

### 2.4. LPAT2 Is Localized to the ER and Catalyzes LysoPA Acylation to PA

**Figure 4:**
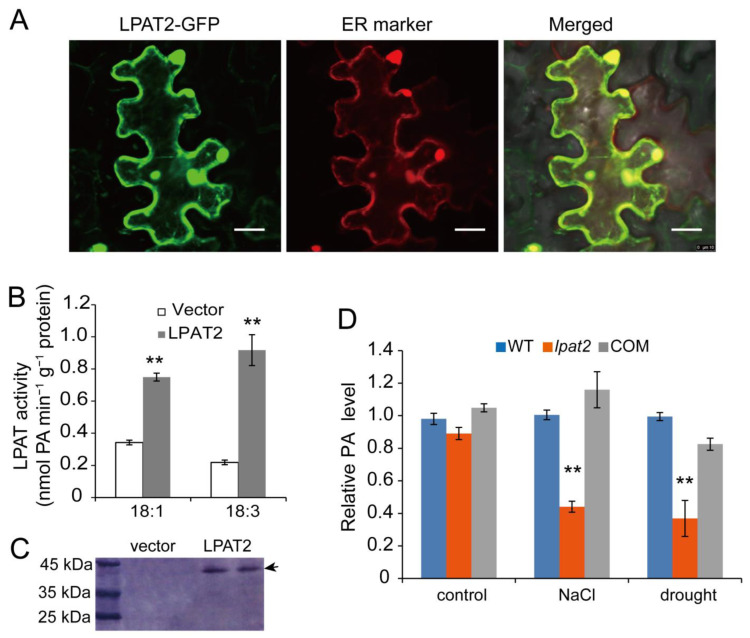
LPAT2 is localized to the endoplasmic reticulum to contribute PA production in vitro and in vivo in osmotic stress. (**A**) LPAT2-GFP is co-localized to the endoplasmic reticulum (ER) marker protein-RFP in tobacco (*Nicotiana benthamiana*) leaves. Bar = 30 μM. (**B**) LPAT2 activity toward lysoPA in the reaction mixtures containing equal amounts of proteins (100 μg) using 18:1-CoA and 18:3-CoA as acyl donors. Values are means ± SD (*n* = 3). ** indicates significant difference at *p* < 0.01 compared with the reaction containing proteins from cells with empty vector control based on Student’s *t* test. (**C**) LPAT2 expression in the *E. coli* strain BL21 cells by Western blotting. The arrow indicates the band related to LPAT2. (**D**) Suppressed LPAT2 led to reduced PA level in plants under salt and drought stress. The lipids were extracted from leaves of three-week-old plants treated with 150 mM NaCl for 12 h or plants withholding water until the relative water content of leaves was 40%. Well-watered plants were used as the control. The lipids were separated on thin layer chromatography (TLC) plate, and relative levels of PA were quantified by using Image J. Values are means ± SD (*n* = 3). ** indicates significant difference at *p* < 0.01 compared with the wild type (WT) based on Student’s *t* test.

In plants, PA is de novo synthesized via two pathways including the eukaryotic pathway in the ER and the prokaryotic pathway in chloroplasts [23]. To investigate whether rice LPAT2 is involved in the prokaryotic or the eukaryotic pathway, the LPAT2 subcellular localization was assessed by fusing LPAT2 with green fluorescent protein (GFP) at the C-terminus transiently expressed in tobacco leaves. Confocal microscopy observation revealed that the GFP fluorescent signal of the LPAT2-GFP overlapped with the ER marker protein (Figure 4A), suggesting that LPAT2 is localized to the ER. Furthermore, to test whether rice *LPAT2* encoded protein has acylation activity, the recombinant LPAT2 with fused His-tagged at the N-terminus was expressed in *E. coli* cells (Figure 4C). The enzymatic assay showed that LPAT2 is capable of catalyzing the LPA acylation to produce PA by using different acyl-CoA species such as 18:1-CoA and 18:3-CoA. A higher activity was found when using 18:3-CoA as an acyl donor (Figure 4B). By contrast, the reaction with proteins extracted from *E. coli* cells containing the empty vector only had relatively low PA production (Figure 4B). The results suggest that LPAT2 catalyzes LPA to produce PA in the ER, which is involved in the PA do novo synthesis via eukaryotic pathway. 

### 2.5. Suppressed LPAT2 Leads to a Substantial Reduction of PA in Plants

The enzymatic activity assay showed that LPAT2 catalyzed LPA to PA. To further test whether suppressed LPAT2 affects PA content in rice plants, lipids were extracted from leaves of *lpat2*, WT, and COM plants treated without (control) and with salt stress or water deficit for lipid quantitative analysis. Under control conditions without salt and water deficit treatments, the PA level in the *lpat2* mutant was slightly lower but not significantly different from that of WT and COM plants (Figure 4D). However, the PA level in the *lpat2* mutant was decreased by ~50%, relative to that of WT when plants were treated with 150 mM NaCl for 12 h. The reduced PA in the *lpat2* mutant was recovered to WT by *LPAT2* complementation (Figure 4D). Likewise, the PA level in the *lpat2* mutant was also significantly lower than that of WT and COM under water deficit condition (Figure 4D). Together, these results suggest LPAT2 contributes PA production in response to salt and water deficit stresses, and that LPAT2 and its product PA may be important for salt and drought stress tolerance in plants.

**Figure 5:**
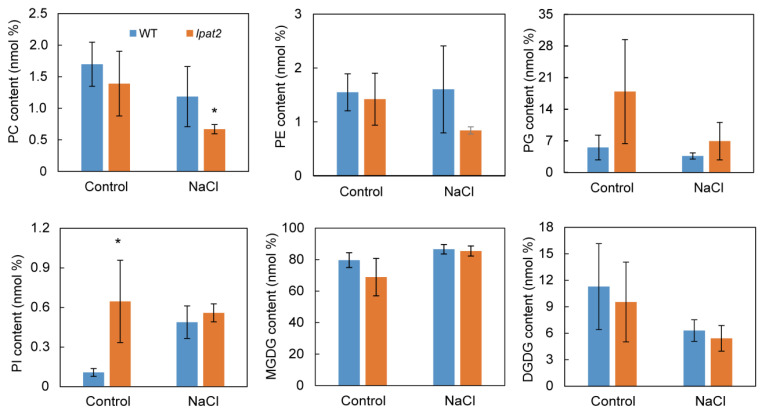
The effect of LPAT2 on glycerolipids in response to salt stress. Total lipids were extracted from leaves of three-week-old plants treated without (control) and with 150 mM NaCl for 12 h. Values are mean ± SE (*n* = 3). * indicates significant difference at *p* < 0.05 compared with the wild type (WT) based on Student’s *t* test.

To test whether suppressed LPAT2 affects other glycerolipids, phospholipids such as phosphatidylcholine (PC), phosphatidylethanolamine (PE), phosphatidylglycerol (PG), and galactolipids were also quantified. Under the control condition, the contents of PC, PE, and PG in the *lpat2* mutant were not significantly different from that of WT, whereas PI content in the *lpat2* mutant was higher than that of WT (Figure 5). However, the PC content in the *lpat2* mutant was significantly lower than that of WT, whereas the PE content in the *lpat2* mutant was lower, but not significantly different from that of WT when plants were treated with 150 mM NaCl for 12 h (Figure 5). The contents of galactolipids such as monogalactosyldiacyglycerol (MGDG) and digalactosyldiacylglycerol (DGDG) were not obviously different between the *lpat2* mutant and WT under both the control and salt stress conditions (Figure 5). The results suggest that suppressed LPAT2 led to reduced PC in some extent in response to salt stress.

### 2.6. Exogenous PA Application in the lpat2 Mutant Rescues Its ABA Sensitivity and Osmotic Stress Tolerance

**Figure 6:**
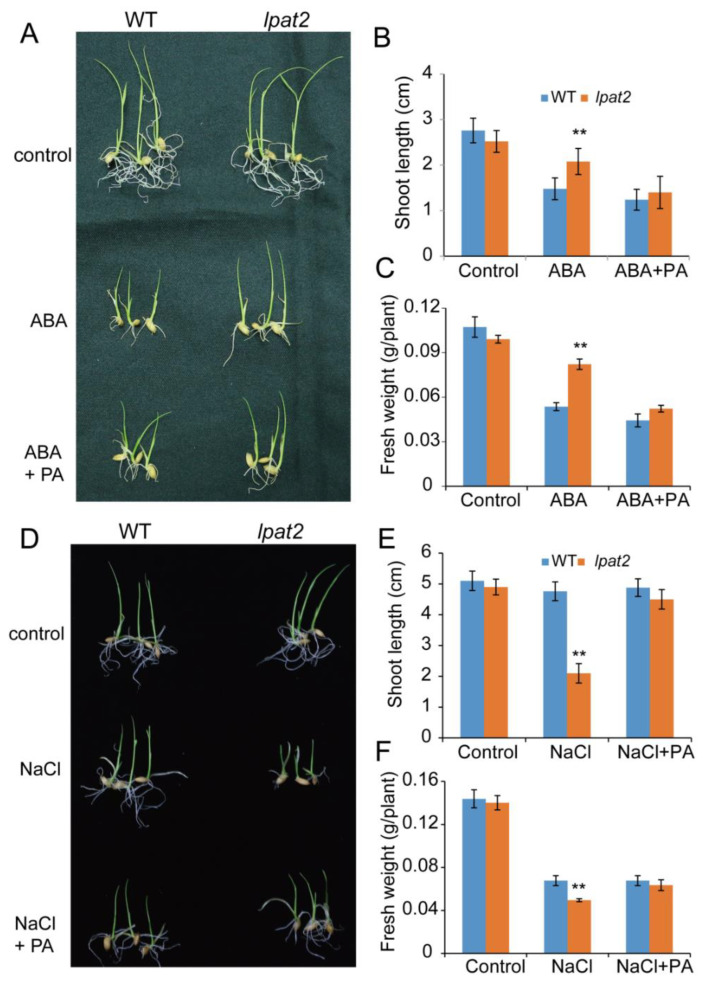
Exogenous PA application rescued the *lpat2* mutant phenotype to WT in response to ABA and salt stress. (**A**–**C**) Exogenous PA application rescued the *lpat2* mutant phenotype to WT in ABA response. Five-day-old seedlings were transferred to liquid medium without supplement (control), with 10 μM ABA, and 10 μM ABA + 20 μg PA for one week. Values are means ± SD (*n* = 10) from one of three independent experiments. (**D**–**F**) Exogenous PA application rescued the *lpat2* mutant phenotype to WT in salt stress response. Five-day-old seedlings were transferred to liquid medium without supplement (control), with 100 mM NaCl, and 100 mM NaCl + 20 μg PA for one week. Values are means ± SD (*n* = 10) from one of three independent experiments. ** indicates significant difference at *p* < 0.01 compared with wild type (WT) based on Student’s *t* test.

The *lpat2* mutant had a lower PA level and less tolerance to osmotic stress as compared to WT plants. To investigate whether LPAT2 itself or LPAT2-derived PA is responsible for ABA signaling and osmotic stress tolerance, the seedlings were transferred to growth medium without or with exogenous PA supplementation under ABA and NaCl treatments. The results showed that the *lpat2* mutant was not significantly different from WT under the control condition without supplementations of ABA and NaCl. However, the *lpat2* mutant seedlings were less sensitive to ABA treatment with longer shoots and greater shoot fresh weight than that of WT (Figure 6A–C). When exogenous PA was applied to the growth medium, the *lpat2* mutant seedlings restored the sensitivity to ABA, and the shoot length and fresh weight of *lpat2* seedlings were rescued to that of WT (Figure 6A–C). By contrast, the *lpat2* mutant had less tolerance to salt stress with shorter shoots and smaller fresh weight than that of WT under salt stress, and the growth inhibition in the *lpat2* mutant was relieved by exogenous PA application (Figure 6D–F). These results suggest that LPAT2-derived PA is required for ABA response and osmotic tolerance.

### 2.7. Suppressed LPAT2 Leads to Altered Expression of Genes Involved in Diverse Processes

**Figure 7:**
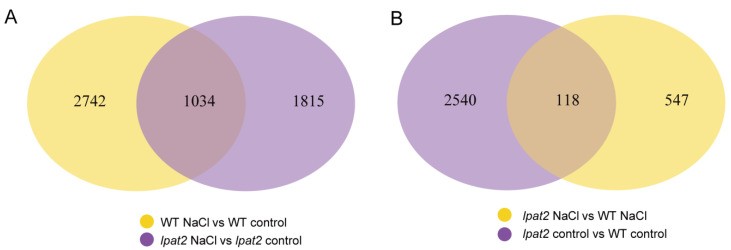

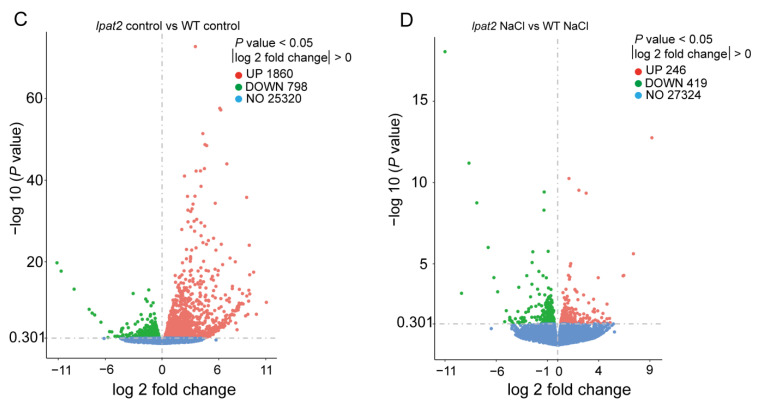
Suppressed LPAT2 led to a large number of differentially expressed genes (DEGs) under control and salt stress conditions. (**A**) The Venn diagram showing the differentially expressed genes (DEGs) of salt stress (100 mM NaCl) versus (vs.) control (without osmotic stress) in wild type (WT) and the *lpat2* mutant. (**B**) The Venn diagram showing the DEGs of the *lpat2* mutant vs. WT under control and salt (100 mM NaCl) stress conditions. (**C**) The numbers of upregulated and downregulated genes of *lpat2* vs. WT under control condition. (**D**) The numbers of upregulated and downregulated genes of *lpat2* vs. WT under NaCl stress condition. Red dots represent upregulated genes and green dots represent downregulated genes. The total RNAs were extracted from leaves of three-week-old plants treated without (control) or with 100 mM NaCl for 12 h for RNA-sequencing.

Phosphatidic acid in a specific membrane region has been shown to bind to various effectors including transcription factors, protein kinases involved in diverse physiological processes such as cell proliferation, cell growth, cell death, vesicular trafficking, hormone response, and abiotic stress responses [19,20,30]. To investigate the effect of LPAT2-derived PA on transcriptome in response to osmotic stress, total RNA was extracted from the leaves of WT and the *lpat2* mutant plants treated without (control) and with 100 mM NaCl for 12 h, for RNA-sequencing. After purification and quality control of mRNA, the differentially expressed genes (DEGs) were identified by comparing the control versus (vs.) salt stress conditions. The number of DEGs in WT was greater than that of the *lpat2* mutant in response to salt stress. A total of 3776 DEGs in WT was found, with 1881 DEGs upregulated and 1895 DEGs downregulated in response to salt stress, whereas that of the *lpat2* mutant was only 2849 with 1172 DEGs upregulated and 1677 DEGs downregulated in response to salt stress (Figure 7A). The results suggest that suppressed LPAT2 leads to decreased DEG number in response to salt relative to control conditions. However, the number of DEGs between *lpat2* vs. WT was 2658 under control conditions, whereas the number of DEGs between *lpat2* vs. WT was 665 under salt stress (Figure 7B–D). The results may indicate that LPAT2 has high impact on overall transcriptional regulation under control conditions without salt stress but the more specific genes were transcriptionally changed in response to salt stress. 

**Figure 8:**
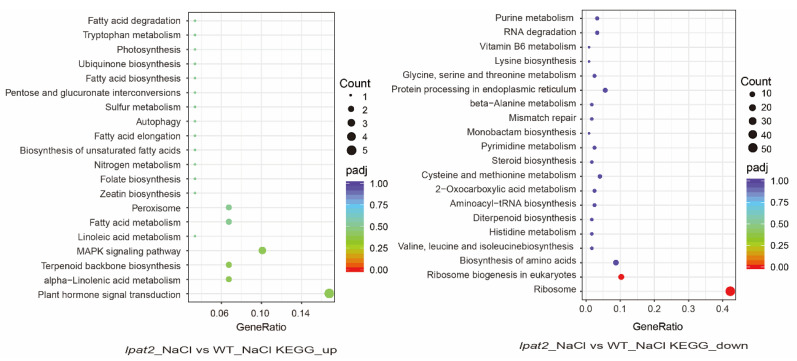
Differentially expressed genes (DEGs) in various pathways by KEGG analysis. Differentially expressed genes (DEGs) in *lpat2* mutant versus (vs) WT under salt stress conditions. The circle size shows the number of DEGs, the color of the circle means p value adjustion (padj), and the horizontal axis means the proportion of DEGs in the total number of genes.

In response to salt stress, upregulated DEGs in the *lpat2* mutant were predominantly involved in plant hormone signaling transduction, MAPK signaling pathway (Figure 8), whereas downregulated DEGs were enriched ribosome and ribosome biogenesis (Figure 8). These results suggest that LPAT2 is important for hormone response and MAPK signaling in osmotic stress and that PA produced by LPAT2 may be a major mediator in these processes.

**Figure 9:**
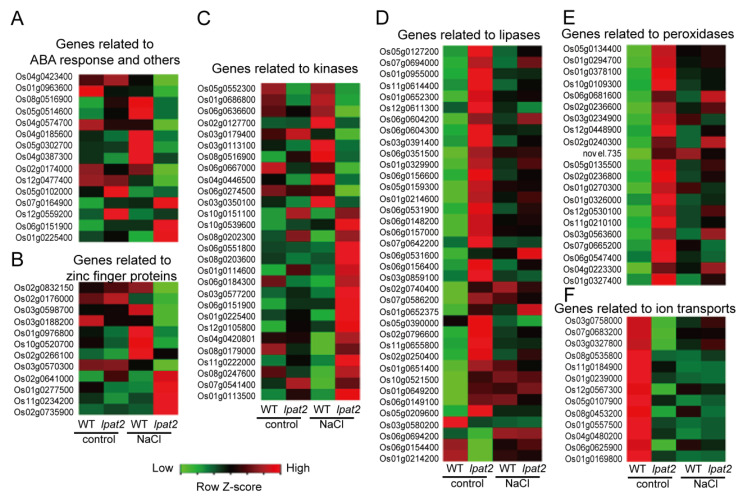
The heat map of selected differentially expressed genes involved in osmotic stress response. Heat map were generated by using the average values of FPKM. Green color shows the downregulation and red shows the upregulation.

To investigate whether altered LPAT2 and its PA affect the expression levels of genes related to osmotic stress, some genes present in DEGs were selected to compare their average values and shown in heat map (Figure 9). The transcript levels of genes involved in ABA response such as ABA-induced proteins (Os04g0423400, Os01g0963600) and SnRK1A protein kinase (Os08g0516900) in the *lpat2* mutant were downregulated under salt stress conditions (Figure 9A). The expression levels of genes related to guard cell anion channel protein (Os04g0574700), phosphate transport (Os04g0185600) and ATP/ADP carrier (Os05g0302700) in the *lpat2* mutant were also downregulated as compared to WT under salt stress conditions (Figure 9A). The expression levels of genes encoding transcription factors in osmotic stress, such as Myb-like (Os02g0174000), NAC (Os12g0477400), and SRF (Os04g0387300), in the *lpat2* mutant were down-regulated relative to WT under salt stress conditions (Figure 9A). However, the expression levels of genes involved in syntheses of ABA (Os07g0164900), jasmonic acid (JA) (Os05g0102000), lipid oxidation (Os12g0559200), and glycolysis (Os06g0151900, Os01g0225400) were upregulated in the *lpat2* mutant relative to WT under salt stress conditions (Figure 9A). Twelve genes encoding zinc finger proteins were altered with eight of them downregulated and four of them upregulated in the *lpat2* mutant relative to WT under salt stress condition (Figure 9B). Moreover, the expression levels of 28 genes related to kinases in the *lpat2* mutants were altered with more promising under salt stress conditions (Figure 9C). In addition, suppressed LPAT2 also led to increased expression levels of genes involved in lipid hydrolysis processes in which include PLD (Os06g0604200, Os06g0604300, Os03g0391400), PLC (Os05g0127200, Os07g0694000, Os01g0955000), phospholipase A (PLA, Os11g0614400, Os01g0652300, Os12g0611300), and GDSL esterases/lipases under control conditions. In particular, the transcript levels of 24 GDSL genes were increased in the *lpat2* mutant relative to WT (Figure 9D). In accordance with induced GDSL genes, the expression levels of 21 peroxidase genes involved in cellular oxidation were also upregulated in the *lpat2* mutant under control conditions (Figure 9E). By contrast, the expression levels of genes encoding NAC transcription factors, Myb transcription factors and ion transports were downregulated in the *lpat2* mutant under control conditions (Figure 9F). Taken together, the results suggest that LPAT2 and its PA play important roles in transcriptional alternations of genes related to ABA response, ion homeostasis, protein kinases in under osmotic stress. In addition, suppressed LPAT2 may lead to increased lipid hydrolysis processes under control conditions.

## 3. Discussion

Of three major sources of PA, PLD-derived PA plays an important role in drought and salt stresses [31,32,34,36]. PLDα1-derived PA enhances stomatal closure under water deficit and ABA treatment in Arabidopsis [31,32,33]. *PLDα3* knockout mutant contains a lower PA level and exhibits susceptibility to salt stress compared to WT plants [34]. The *pldα1*/*δ* double mutant plants with reduced PA level are more sensitive to salt stress than that of WT and the single mutant [35,36]. Phosphatidic acid produced from PLC/DGK also plays a role in osmotic stress response [38,39]. However, whether the de novo synthesized PA is involved in osmotic stress remains unknown. LPAT catalyzes a committed step in PA de novo synthetic pathway [25,26]. In Arabidopsis, LPAT1 is involved in the prokaryotic pathway of PA synthesis and is essential for embryo development [25,28]. Of the remaining four LPATs involved in the eukaryotic pathway, LPAT2 is most abundant and the *LPAT2* knockout mutant is lethal at the female gametophyte developmental stage [26]. By comparison, the single mutant for *LPAT3*, *LPAT4,* or *LAPT5* exhibits no overt phenotype [27]. These results suggest that LPAT2 is a primary LPAT in the eukaryotic pathway. Recent studies showed that LPAT2 is also involved in phosphate starvation [29], whereas LPAT4 and LPAT5 function redundantly in nitrogen starvation response [27]. Phosphatidic acid is an important signal molecule in osmotic stress [18,20]. However, the role of LPAT-derived PA in salt and drought stress is still unclear. In this study, we isolated a knockdown mutant for *LPAT2* in rice that exhibited less performance in osmotic tolerance, accompanied by a reduced PA level under salt and water deficit conditions. Supplemented exogenous PA in growth medium recovered the osmotic sensitive phenotype of the *lpat2* mutant to WT. Moreover, genetic complementation by *LPAT2* was able to restore PA level of the *lpat2* mutant to WT plants, and hence recovered the *lpat2* mutant phenotype to WT. Thus, the results suggest that PA produced by LPAT2 activity plays an important role in salt and drought stress response in rice. 

Abscisic acid is a key phytohormone in promoting stomatal closure to prevent water loss under salt stress and water deficit [2,40]. Two key players, snRK2 and ABI1 (PP2C), exhibit opposite actions in ABA signaling. In the absence of ABA, SnRK2 functions are restricted by ABI1, whereas upon osmotic stress, ABA is produced and binds to ABA receptor PYR1/PYL to relieve ABI1 inhibition. Subsequently SnRK2 was activated to phosphorylate the downstream substrates, thereby promoting stomatal closure to reduce water transpiration [15]. PLD-derived PA is known to play an important role in ABA signaling. PLDα1-derived PA is required for the effects of ABA on stomatal movement through interaction with ABI1 (PP2C), a negative regulator of ABA responses [31,32]. Moreover, PLDα1-derived PA binds to NADPH oxidase to mediate ABA-induced reactive oxygen species (ROS) and nitric oxide (NO) in guard cells in Arabidopsis [41]. Meanwhile, PA is accumulated and increases ABA sensitivity to regulate seed germination [42]. PLD-derived PA can mimic the effects of ABA, including the inhibition of α-amylase production in aleurone cells and suppression of seed germination [43]. A reduced PA resulted from double knockout mutation of *PLDα*1 and *PLDδ* renders seeds less sensitive to ABA inhibition of seed germination [44]. Our results showed that LPAT2-derived PA in osmotic stress tolerance is mediated by ABA response, as reduced ABA sensitivity of the *lpat2* mutant was restored to WT by either *LPAT2* genetic complementation or exogenous PA application. The results suggest that PA derived from different sources may exhibit similar function in osmotic stress response. Further studies are needed to investigate how LPAT2-derived PA mediates ABA response. 

PA functions as a signal at upstream, a small change of PA level may lead to a significant impact on physiological effects [20]. Transcriptomic analysis revealed that suppressed LPAT2 led to a large number of DEGs under salt stress. In response to salt stress, downregulated DEGs in the *lpat2* mutant included genes related to ABA response, Myb and NAC transcript factors, protein kinases, zinc finger proteins, transports of ions and ATP. By contrast, the expression levels of genes involved in the syntheses of ABA, JA, and glycolysis, were upregulated in the *lpat2* mutant relative to WT under salt stress. The results suggest that LPAT2 affects the transcript levels of genes responsible for ABA signaling, hormone synthesis, ion transports, and protein kinases. It was shown that protein kinases play central roles in abiotic stress in plants [2]. PLD-derived PA interacts with mitogen-activated protein kinase 6 (MPK6) to regulate salt stress tolerance [45]. Our results showed that many genes encoding protein kinases were transcriptionally altered with most downregulated in *LPAT2*-suppressed mutant. The results suggest that LPAT2-derived PA has an effect on the transcript levels of protein kinases under salt stress. It was shown that stress-activated protein kinases such as SnRK2s play important roles in transcriptional regulation through the phosphorylation of the transcription factor [46]. PA is an important mediator in ABA signaling. Most DEGs are probably caused by the indirect effects of LPAT2-derived PA. Nevertheless, PA was found to interact with several transcription factors in different cellular processes. PLDζ-derived PA binds to werewolf (WER), a R2R3 MYB transcription factor (TF), to module root hair formation and elongation [47]. PA interacts with two MYB TFs, late elongated hypocotyl (LHY) and circadian clock associated 1 (CCA1), to regulate lipid metabolism and seed oil accumulation [48]. PA also binds to AT-hook motif-containing nuclear localized (AHL) transcription factor to regulate triacylglycerol (TAG) degradation and seedling growth [49]. It is possible that LPAT2-derived PA is also involved in transcriptional regulation through binding to a new transcription factor in osmotic stress response. It will be of interest to identify new target(s) of LPAT2-derived PA that is directly responsible for transcriptional regulation in response to salt and water deficit stress in future studies.

## 4. Materials and Methods 

### 4.1. Plant Growth and Treatments

The rice mutant and the genetic complementation (COM) were derived from the rice Zhonghua 11 (*Oryza sativa* L.). Wild type (WT) plants with same genetic background were used as a control. Rice plants were grown in the experimental field of Huazhong Agricultural University (Wuhan, China) under natural conditions during the seasons of summer and fall for genotype identification and seed collection. For stress treatments, seven-day-old seedlings were transferred to the pots containing soil (paddy soil:histosols = 1:1, *v*/*v*) and watered regularly until the plants were ready for treatments. For salt treatments, three-week-old plants were treated with various concentrations (0, 100, 150 mM) of NaCl solution for various time points in growth room under the conditions of 14 h light (30 °C)/10 h dark (26 °C), photosynthetic photon flux density of 450–600 μmol·m^−2^·s^−1^, and 50% relative humidity or under natural conditions during summer/fall seasons. For water deficit treatment, watering was stopped for three-week-old plants until leaves of WT plants began wilting when the soil moisture content was approximately 11% in the growth room under the conditions shown above. The soil moisture content was measured according to the method described by Mishra et al. [50]. Briefly, fresh soil was sampled from the pots under the control and water deficit conditions. The soil was weighed (W1) and dried at 60 °C in an oven. After drying, the soil was reweighed (W2) and moisture content was calculated by the following formula: % soil moisture = 100 × ((W1 − W2)/W2). For PA and ABA treatments, the seeds were germinated in small plastic cups supplemented with 10 μM ABA, 100 mM NaCl, 10% PEG without or with 20 μg PA based on previous methods [21,34,38]. Seed germination rate was scored by taking radical emergence as a criterion for germination.

### 4.2. Phylogenetic Analysis

The phylogram was constructed based on the method described previously [51]. The amino acid sequences of rice, Arabidopsis, maize, soybean, algae and yeast LPATs were obtained from Rice Genome Annotation Project (http://rice.plantbiology.msu.edu/, accessed on 3 March 2010), Arabidopsis Information Resource (http://www.arabidopsis.org/, accessed on 3 March 2010), Saccharomyces GENOME DATABASE (https://www.yeastgenome.org/, accessed on 28 June 2022), MaizeGDB (https://www.maizegdb.org/, accessed on 28 June 2022), SoyBase (https://www.soybase.org/, accessed on 28 June 2022) and Uniport (https://www.uniprot.org/, accessed on 28 June 2022) databases, respectively. *Selaginella* LPATs protein sequences were searched by BLASTP on NCBI by using *Arabidopsis thaliana* LPATs protein sequences. Amino acid sequence alignment was analyzed by using MAFFT software (7.490, Research Institute for Microbial Diseases, Osaka University, Suita, Japan). The phylogenetic was constructed by using Mega 11 (https://www.megasoftware.net/, accessed on 28 June 2022) software, with Neighbor-Joining method, Poisson model and 1000 Bootstrap tests. The phylogenetic analysis was embellished by EvolView (http://www.evolgenius.info/evolview/, accessed on 28 June 2022).

### 4.3. RNA Extraction and Quantitative Real Time PCR

Total RNA was extracted from various tissues by using Trizol reagent (TransGene Biotech, Beijing, China). The contaminated DNA was removed by RNase free DnaseI. The first strand cDNAs were synthesized from mRNA templates by using TransScript cDNA Synthesis SuperMix kit (Trans-Gene Biotech, Beijing, China). The cDNA concentration was adjusted to a similar level based on the Ct value for *β-actin* (LOC_Os01g16414) by quantitative real-time PCR. The resultant cDNAs were used for quantitative real-time PCR monitored by a MyIQ real time PCR system (Bio-Rad, Hercules, CA, USA) by using Top Green qPCR SuperMix (TransGene Biotech, Beijing, China). Real-time PCR was run under the following conditions: 95 °C for 1 min; 40 cycles of 9 5°C for 30 s, 55 °C for 30 s, 72 °C for 30 s. The primers for quantitative real-time PCR are listed in Supplementary Table S1.

### 4.4. Mutant Isolation and Genetic Complementation 

A T-DNA insertion mutant in *LPAT2*, designated *lpat2*, was identified from the T-DNA insertion collections, and the mutant seeds were obtained from the Huazhong agricultural University (RMD; http://rmd.ncpgr.cn/, accessed on 3 March 2010). The homozygous mutant *lpat2* was isolated by using the T-DNA left border primer coupled with *LPAT2*-specific primers. For genetic complementation of the *lpat2* mutant, the native *LPAT2* promoter (2000 bp) was amplified from rice genomic DNA by using primers COMF and COMR with cutting sites of *EcoR* I and *Kpn*I. The *LPAT2* CDS was cloned from cDNA synthesized from rice leaf mRNA by using LPAT2 FK and LPAT2 Rb primers containing cutting sites of *Kpn*I and *BamH*I. The resulting fragments were ligated to the pCAMBIA2301 vector. The construct was introduced into *Agrobacterium tumefaciens EHA105*, which was used to infect the *lpat2* mutant calli to generate *LPAT2* genetic complementation plants. The primers for real-time PCR are listed in Supplementary Table S1.

### 4.5. Measurements of Ion Leakage and Malonaldehyde

For ion leakage, the leaves of the same size, position, and age were excised from plants. After washing briefly with deionized water, the leaf samples were immersed in 15 mL distilled water in glass tubes. Gas was removed from the leaf surface in a vacuum for 30 min subsequently and incubated with gentle agitation for 3 h. The initial conductivity was measured by using a conductivity meter, and then the samples were boiled in a water bath for 20 min. Total conductivity was measured again after cooling to room temperature. Ion leakage was calculated based on a percentage of the initial conductivity versus the total conductivity.

The malonaldehyde (MDA) content of rice leaves was measured based on Zhao et al. [52]. Briefly, leaves (0.5 g per sample) were homogenized and incubated with 10% (*w*/*v*) trichloroacetic acid containing 0.6% (*w*/*v*) thiobarbituric acid at 95 °C for 30 min. The solution was cooled to room temperature and centrifuged at 12,000× *g* for 5 min. The absorbance of the supernatant was measured at 450, 532, and 600 nm, respectively. The MDA content was calculated according to the following equation: 6.45 × (OD_532_ − OD_600_) − 0.559 × OD_450_.

### 4.6. Subcellular Localization

To investigate the subcellular localization of LPAT2, the coding sequence of LPAT2 was cloned into the pMDC83 vector in-frame with the C-terminal fusion to GFP. The construct was transformed into *Agrobacterium tumefaciens* (EHA105) and the transformed *Agrobacterium* was grown in liquid LB media until OD_600_ reached 0.6, and centrifuged at 4000 rpm for 10 min. The cells were resuspended in the solution (50 mM MES pH 5.7, 10 mM MgCl_2_, 150 mg/mL acetosyringone) and infiltrated the leaves of four-week-old tobacco (*Nicotiana benthamiana*) [53]. Five days after infection, GFP fluorescence was observed with a Lecia TCS SP2 confocal microscope with GFP excited at 488 nm and imaged at 500 to 530 nm, and RFP excited at 540 nm and imaged at 575 to 625 nm.

### 4.7. LPAT2 Expression and Activity Assay

The full-length of *LPAT2* CDS was amplified by PCR from cDNA that was synthesized from mRNA of rice leaves by using LPAT2-ProF and LPAT2-ProR primers containing *EcoR*I and *Xho*I cutting sites (Supplementary Table S1). The CDS was ligated in frame into the pET28a expression vector. The resultant construct containing *LPAT2* was transformed into *Escherichia coli* BL21 cells for LPAT2 expression. The LPAT2 activity was assayed in a reaction mixture (50 mM Tris HCl, pH 7.5, 0.5 M MgCl_2_, lysoPA, acyl-CoA) in a final volume of 100 μL with 100 μg proteins. After incubation at 37 °C for 1 h, the reaction was stopped by addition of 1 mL of chloroform/methanol (*v*/*v*, 2:1) and 250 μL of 1 N HCl. The organic phase was dried under a stream of nitrogen and dissolved in 20 μL of chloroform. The resultant lipids of reaction were separated on a thin-layer chromatography (TLC) plate (Merck, TLC silica gel 60) by developing a solvent of petroleum ether: diethyl ether: acetic acid (50:50:1, *v*/*v*). Finally, TLC plate was exposed to iodine gas to visualize PA. The spots corresponding to PA were quantified by ImageJ software (1.48, National Institutes of Health, Bethesda, MD, USA).

### 4.8. Lipid Extraction and Analysis

Lipids were extracted as a described method [54]. Briefly, leaves were sampled and immediately immersed in 5 mL of isopropanol containing 0.01% butylated hydroxytoluene (BHT, Sigma-Aldrich, St. Louis, MO, USA) at 75 °C for 15 min. After cooling to room temperature, the solvent (3.5 mL) of chloroform/water (2.5:1, *v*/*v*) was added to the sample and incubated for 1 h at room temperature by shaking. The extracts were transferred to clean glass tubes, and then the remaining samples were re-extracted with 5 mL chloroform/methanol (2:1, *v*/*v*) containing 0.01% BHT at room temperature for several times until leaf tissues became bleached. The extracts from the same sample were combined and washed twice with 1 M KCl and once with water, and dried under a stream of nitrogen gas, and then dissolved in a defined volume of chloroform for analysis by TLC combined with GC measurement. For glycerolipid quantification, the lipids were separated on TLC by using the developing solvent composed of acetone, toluene, and water (91:30:7.5, *v*/*v*) [55]. The spots in the TLC plate visualized with iodine were scraped out and methylated in methanol containing 1% H_2_SO_4_ and 0.05% BHT at 80 °C for 30 min, and then quantitatively analyzed by GC measurement by using glyceryl triheptadecanoate (Sigma-Aldrich, St. Louis, MO, USA) as the internal standard. The GC was run under the following conditions. The temperature for the injection port was set at 180 °C with the pressure at 16.06 psi and the split ratio of 20:1. The oven temperature was initiated at 180 °C for 2 min and then was increased by 10 °C/min up to 220 °C for 5 min. The temperature of the flame ionization detector is 280 °C with the flow rate of 30, 300, and 25 mL/min for hydrogen, air, and helium, respectively. PA was detected based on previous methods [56]. Briefly, the TLC plates were dried in an oven at 110 °C for 1 h and then cooled to room temperature. The lipids were separated on TLC by using developing solvent (chloroform: ethanol: triethylamine: water = 10:11.3:11.7:2.7, *v*/*v*). The spots corresponding PA were visualized with 0.4% bromothymol blue solution containing 10 mM NaOH and quantified by ImageJ software (1.48, National Institutes of Health, USA).

### 4.9. RNA Sequencing and Analysis

The quality and integrity of RNA extracted from leaves were assessed by using the RNA Nano 6000 Assay Kit of the Bioanalyzer 2100 system (Agilent Technologies, Santa Clara, CA, USA). A total RNA (1 μg RNA/sample) was used as input material for the RNA sample preparations. Briefly, mRNA was purified from total RNA by using poly-T oligo-attached magnetic beads and was fragmentated by using divalent cations under elevated temperature. First strand cDNA was synthesized by using random hexamer primer and M-MuLV reverse transcriptase (RNase H-) and the second strand cDNA was synthesized by using DNA polymerase I and RNase H. The library fragments with preferential length of 370~420 bp were selected by AMPure XP system (Beckman Coulter, Beverly, MA, USA). The library quality was assessed on the Agilent Bioanalyzer 2100 system. Sequencing of cDNA was performed on an Illumina Novaseq platform. The read number of a gene was counted by using FeatureCounts v1.5.0-p3. The fragments per kilobase of transcript per million mapped reads (FPKM) of a gene was calculated based on the length of this gene. Differential expressed gene (DEG) analysis was performed by using the DESeq2 R package (1.20.0). The resulting *p*-values were adjusted by using the Benjamini and Hochberg approach for controlling the false discovery rate. Genes with an adjusted *p*-value < 0.05 found by DESeq2 were assigned as differentially expressed.

## Supplementary Materials

The following supporting information can be downloaded at: https://www.mdpi.com/article/10.3390/ijms23179796/s1.
