# Supplementary material for: The Examination of the Role of Rice Lysophosphatidic Acid Acyltransferase 2 in Response to Salt and Drought Stresses
Source: Int J Mol Sci. 2022 Aug 29;23(17):9796. doi: 10.3390/ijms23179796 (PMC9456497; doi:10.3390/ijms23179796)
Supplement: Supplementary file 1 [file ijms-23-09796-s001.zip › ijms-1861485-supplementary.pdf]

Supplementary Table S1. Primers used in this study.

Primer name	Primer sequence (5'→3')	Primer Specification
FP	TTATAGGTGCCCCAAAACCC	*lpat2* mutant identification
RP	TTACAGTTTCGCGCAGTGAC	*lpat2* mutant identification
T-border primer	ATTCCAGATCCCCCGAATTA	*lpat2* mutant identification
COMF	GAATTCGCGAAAAACGCACCGTATAG	Promoter for LPAT2 COM
COMR	GGTACCATCTTTCTTGACACGGTTC	Promoter for LPAT2 COM
LPAT2 FK	GGTACCATGGCGGTCCCACTCGTGC	CDS for *LPAT2* COM
LPAT2 Rb	GGATCCATCTTTCTTGACACGGTTC	CDS for *LPAT2* COM
Pcambia-F	TAGCCAATACGCAAACCGC	COM identification
OsLp2	CTTCAGTAACCCTTGGGCATAG	COM identification
LPAT2-ProF	GAATTCATGGCGGTCCCACTCGTGC	LPAT2 protein expression
LPAT2-ProR	CTCGAGATCTTTCTTGACACGGTTCC	LPAT2 protein expression
LPAT2-GFPF	GGGGTACCATGGCGGTCCCACTCGTGC	LPAT2-GFP localization
LPAT2-GFPR	GCTCTAGAATCTTTCTTGACACGGTTCC	LPAT2-GFP localization
LPAT2-RTF	AGTGAGATGCCAAAGTCA	Real time PCR
LPAT2-RTR	ACAGGAGTTGAGTCCATAGG	Real time PCR
β-actin F	TGCTATGTACGTCGCCATCCAG	Real time PCR
β-actin R	AATGAGTAACCACGCTCCGTCA	Real time PCR

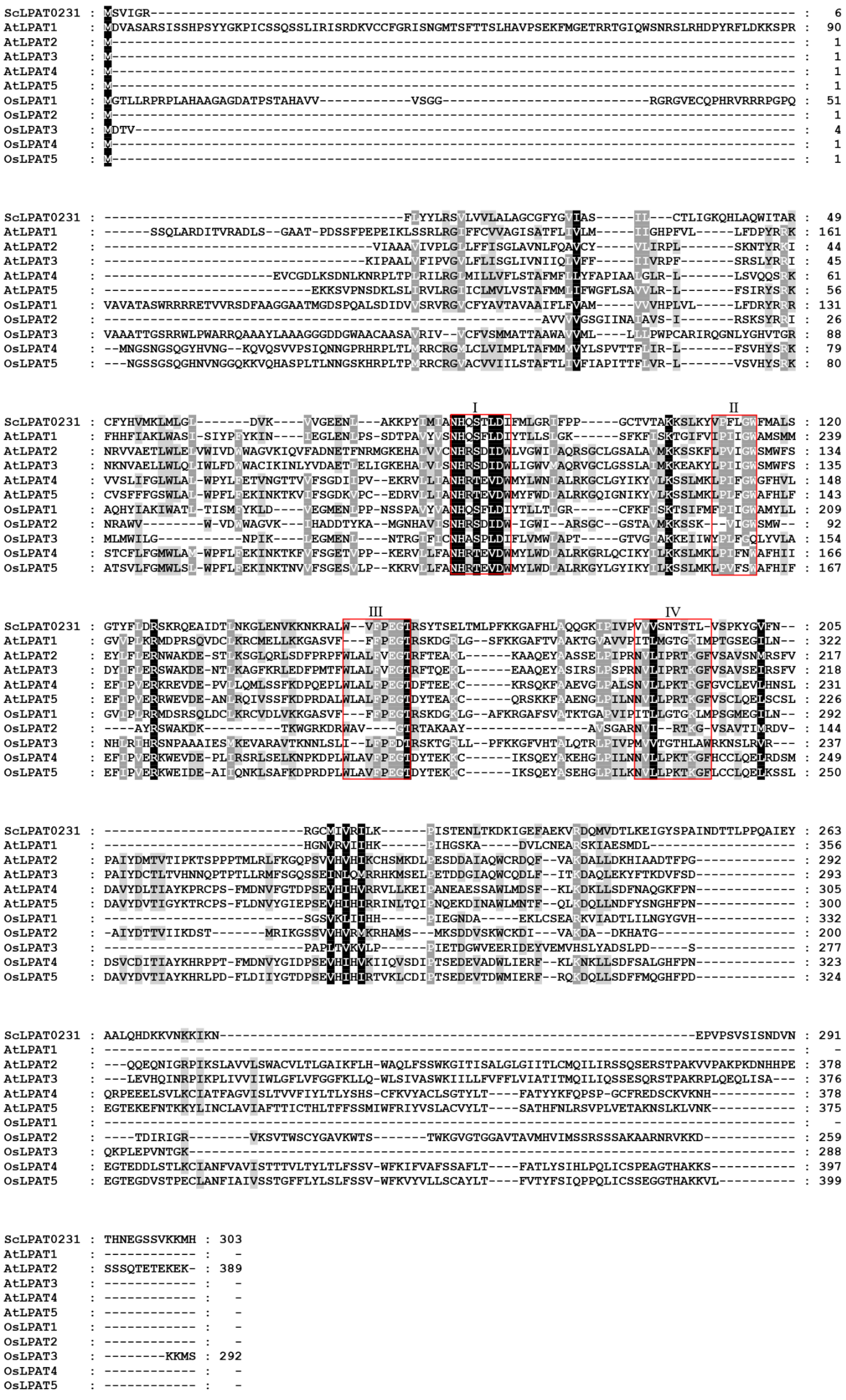

Supplementary Figure S1. Sequence alignment and domain structures of LPATs.

Alignment of amino acid sequences of LPATs were analyzed using MAFFT. The identical and similar residues are shaded in black and gray, respectively. The conserved motifs including I, II, III and IV related to acyltransferases are indicated in boxes. Sc, *Saccharomyces cerevisiae;* At, *Arabidopsis thaliana;* Os, *Oryza sativa.*

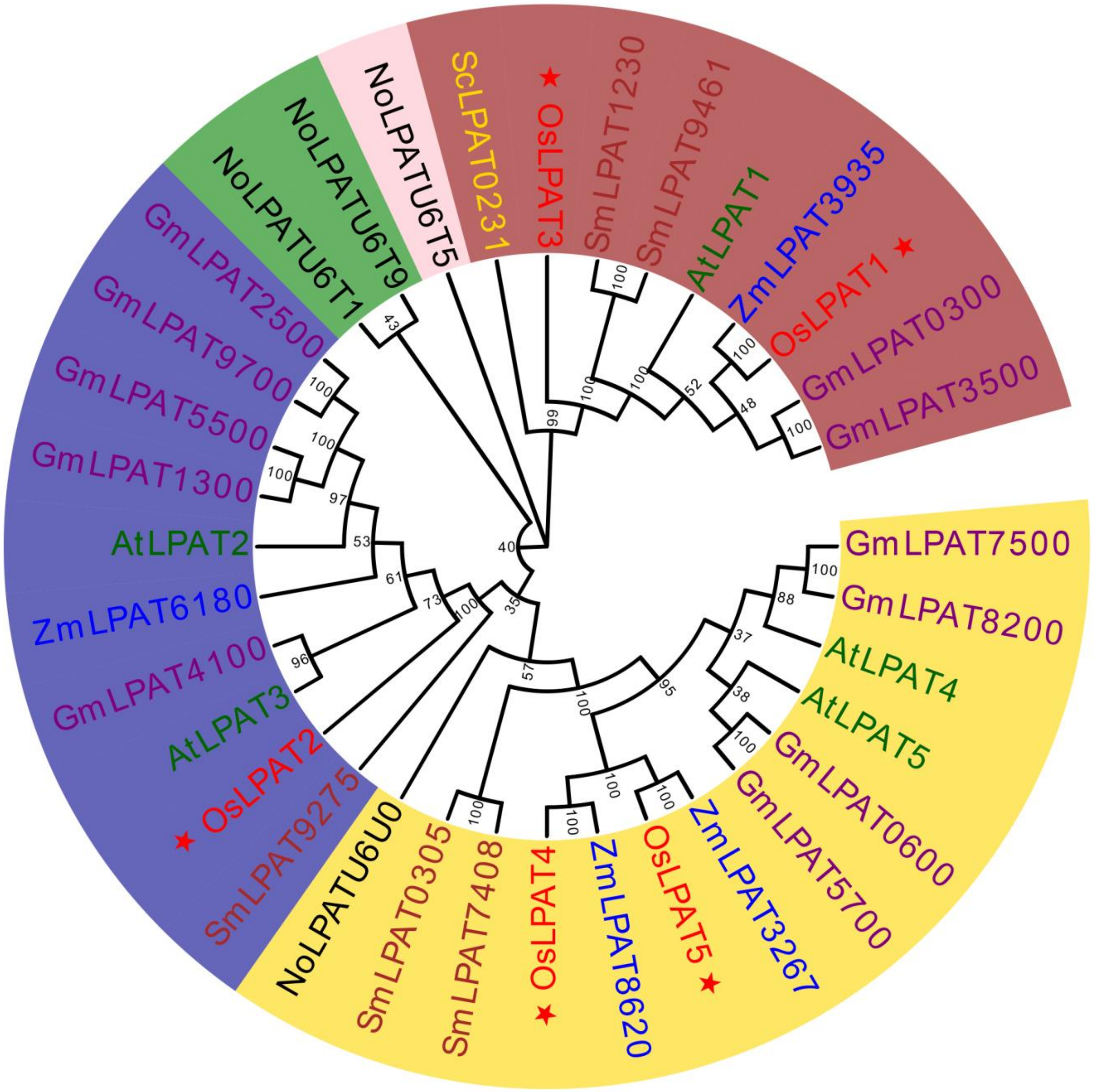

Supplementary Figure S2. Phylogenetic analysis of LPAT family in various species.

The amino acid sequences of LPAT orthologous proteins were aligned using the MAFFT software and The phylogenetic tree was constructed through MEGA11 software using neighbor-joining and bootstrap (1000) as default setting. At, *Arabidopsis thaliana*; Gm; *Glycine max*; Os, *Oryza sativa*; Zm, *Zea mays*; Sm, *Selaginella moellendorffii*; No, *Nannochloropsis oceanica*; Sc, *Saccharomyces cerevisiae.* OsLPAT1, Os10g0497100; OsLPAT2, Os11g0637800; OsLPAT3, Os04g0625200; OsLPAT4, Os05g0502200; OsLPAT5, Os01g0782500; AtLPAT1, At4g30580; AtLPAT2, At3g57650; AtLPAT3, At1g51260; AtLPAT4, At1g5020; LPAT5, At4g18850.
